# Integrating somatic CNV and gene expression in breast cancers from women with *PTEN* hamartoma tumor syndrome

**DOI:** 10.1038/s41525-023-00361-0

**Published:** 2023-07-05

**Authors:** Takae Brewer, Lamis Yehia, Peter Bazeley, Charis Eng

**Affiliations:** 1 Genomic Medicine Institute, Lerner Research Institute, Cleveland Clinic, Cleveland, OH 44195 USA; 2 Department of Molecular Medicine, Cleveland Clinic Lerner College of Medicine, Case Western Reserve University, Cleveland, OH 44195 USA; 3 Department of Quantitative Health Sciences, Lerner Research Institute, Cleveland Clinic, Cleveland, OH 44195 USA; 4 Taussig Cancer Institute, Cleveland Clinic, Cleveland, OH 44195 USA; 5 Department of Genetics and Genome Sciences, Case Western Reserve University School of Medicine, Cleveland, OH 44106 USA; 6 Germline High Risk Cancer Focus Group, Case Comprehensive Cancer Center, Case Western Reserve University, Cleveland, OH 44106 USA

**Keywords:** Cancer genomics, Molecular medicine, RNA sequencing, DNA sequencing, Breast cancer

## Abstract

Women with germline *PTEN* variants (*PTEN* hamartoma tumor syndrome, PHTS) have up to 85% lifetime risk of female breast cancer (BC). We previously showed that PHTS-derived BCs are distinct from sporadic BCs both at the clinical and genomic levels. In this study, we examined somatic copy number variations (CNV) and transcriptome data to further characterize the somatic landscape of PHTS-derived BCs. We analyzed exome sequencing data from 44 BCs from women with PHTS for CNV. The control group comprised of 558 women with sporadic BCs from The Cancer Genome Atlas (TCGA) dataset. Here, we found that PHTS-derived BCs have several distinct CNV peaks compared to TCGA. Furthermore, RNA sequencing data revealed that PHTS-derived BCs have a distinct immunologic cell type signature, which points toward cancer immune evasion. Transcriptomic data also revealed PHTS-derived BCs with pathogenic germline *PTEN* variants appear to have vitamin E degradation as a key pathway associated with tumorigenesis. In conclusion, our study revealed distinct CNV x transcript features in PHTS-derived BCs, which further facilitate understanding of BC biology arising in the setting of germline *PTEN* mutations.

## Introduction

Phosphatase and tensin homolog (*PTEN*), a tumor suppressor gene^1^, is one of the most frequently somatically altered genes in different malignancies including breast cancer (BC)^2^. *PTEN* hamartoma tumor syndrome (PHTS) encompasses individuals harboring a germline *PTEN* variant, which causes heritable predisposition to specific cancers including breast, thyroid, kidney, endometrial and colon cancers, and melanoma^3^. PHTS-derived BCs have distinct clinical characteristics compared to sporadic counterparts. Women with PHTS have up to 85% lifetime risk of breast cancer (BC), which is notably higher than that in the general population (12.9% lifetime risk)^3^. Furthermore, women with PHTS have a much younger onset of BC diagnosis, as well as a significantly higher incidence of second primary BC^4^.

PHTS-derived BCs are distinct not only at the clinical but also at the molecular and genomic levels. Recently, we found that BCs arising in the setting of PHTS had a distinct somatic mutational landscape compared to that of their sporadic counterparts^5^. We demonstrated that PHTS-derived BCs had a high frequency of somatic second hits to the *PTEN* gene (where the underlining germline *PTEN* variants represent the first hit), which appeared to be driving carcinogenesis. Furthermore, BCs from PHTS patients with germline pathogenic or likely pathogenic *PTEN* variants (Tier-1 variants), had much fewer somatic mutations in *PIK3CA* compared to those in TCGA and in PHTS-Tier 2 (variant of unknown significance or likely benign variants) BCs. Our findings were consistent with the observation that the nature of the underlying germline mutations in cancer tissues influences somatic phenotypes^6^.

BC biology and its genomic landscape are complex and need to be understood in the context of large genomic and functional genomic changes such as somatic copy number variation (CNV) and gene expression differences^7,8^. In this study, we further characterized the somatic landscape of PHTS-derived BCs by examining somatic CNVs and the transcriptome.

## Results

### Somatic CNV analysis of PHTS and TCGA BC

**Fig. 1:**
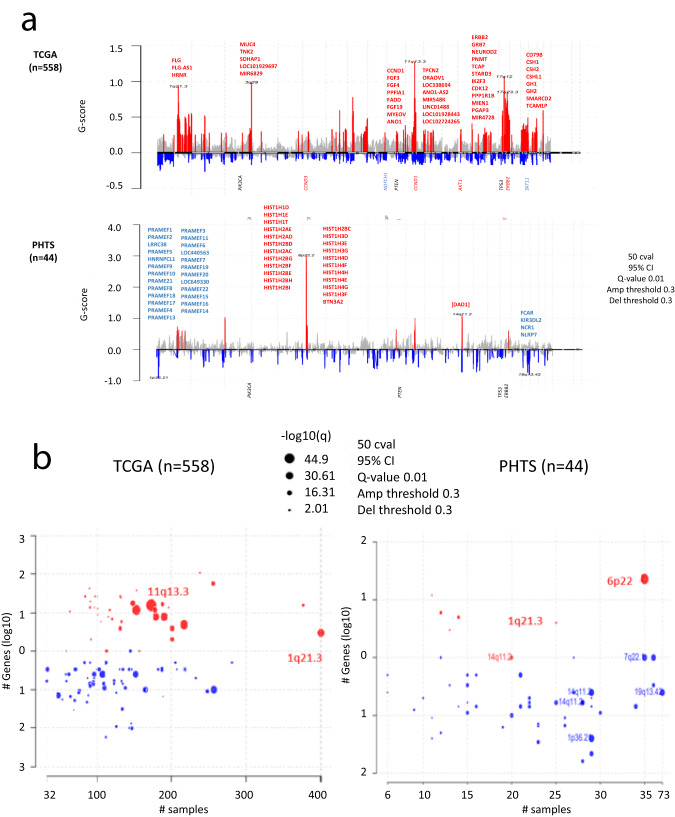
Recurrent CNV peaks and altered cytobands in PHTS-derived BCs and sporadic BCs from TCGA. **a** Genome plots showing recurrent CNV peaks identified by gistic2 in samples from TCGA (top) and from PHTS-derived BCs (bottom). Red peaks represent recurrent amplification and blue, deletion peaks. Genes contained in some of the significant peak regions are shown. Positions of BC-associated genes are shown at the bottom of each plot, shown in red if an amplification is present and in blue if a deletion is observed. **b** Gistic bubble plots showing significantly altered cytobands in BC samples from TCGA (left panel) and in PHTS-derived BCs (right panel). The *X*-axis represent the number of samples which had CNV alterations and the Y-axis, the number of genes each bubble contains. The bubble size is according to −log10 transformed *q* values.

We identified seven significant amplification peaks and 46 significant deletion peaks in PHTS-derived BCs (Fig.1a, Supplementary Table 2, Supplementary Table 3). In TCGA BCs, there were 37 amplification peaks and 63 deletion peaks. Four out of seven CNV amplifications (3p26.1, 6p22.2, 10q21.2, 11q13.1) are present in PHTS-derived BC samples but not in sporadic BC samples from TCGA. The most significant peak in this group is at 6p22.2 (Fig. 1b), which was absent in TCGA. This region contains multiple histone-related genes including *HIST1H1*, *HIST1H2*, *HIST1H3*, and *HIST1H4* families (Supplementary Table 4). Nine out of 36 samples (25.0%) with significant amplifications at 6p22.2 had somatic *PTEN* variants which were distinct from their respective germline *PTEN* variants, while only one out of eight samples (12.5%) without a 6p22.2 CNV amplification peak had a somatic *PTEN* hit. This difference did not reach significance (odds ratio [OR] 2.33, 95% CI 0.29–28.9, *p* = 0.66).

For deletion peaks, 28 out of 46 regions were present in PHTS BCs but absent in TCGA BCs (Supplementary Table 3). No amplification or deletion peaks in PHTS-derived BCs contained any of the 82 BC-associated genes (Supplementary Table 1) including *ERBB2*, *EGFR*, *PTEN*, and *TP53* (Supplementary Table 4). In contrast, amplification peaks containing *CCND3* (6p21.1), *CCND1* (11q13.3), *AKT1* (14q32.33), and *ERBB2* (17q12), and deletion peaks containing *NOTCH1* (9q34.3) and *STK11* (19p13.3) were identified in TCGA BCs.

**Fig. 2:**
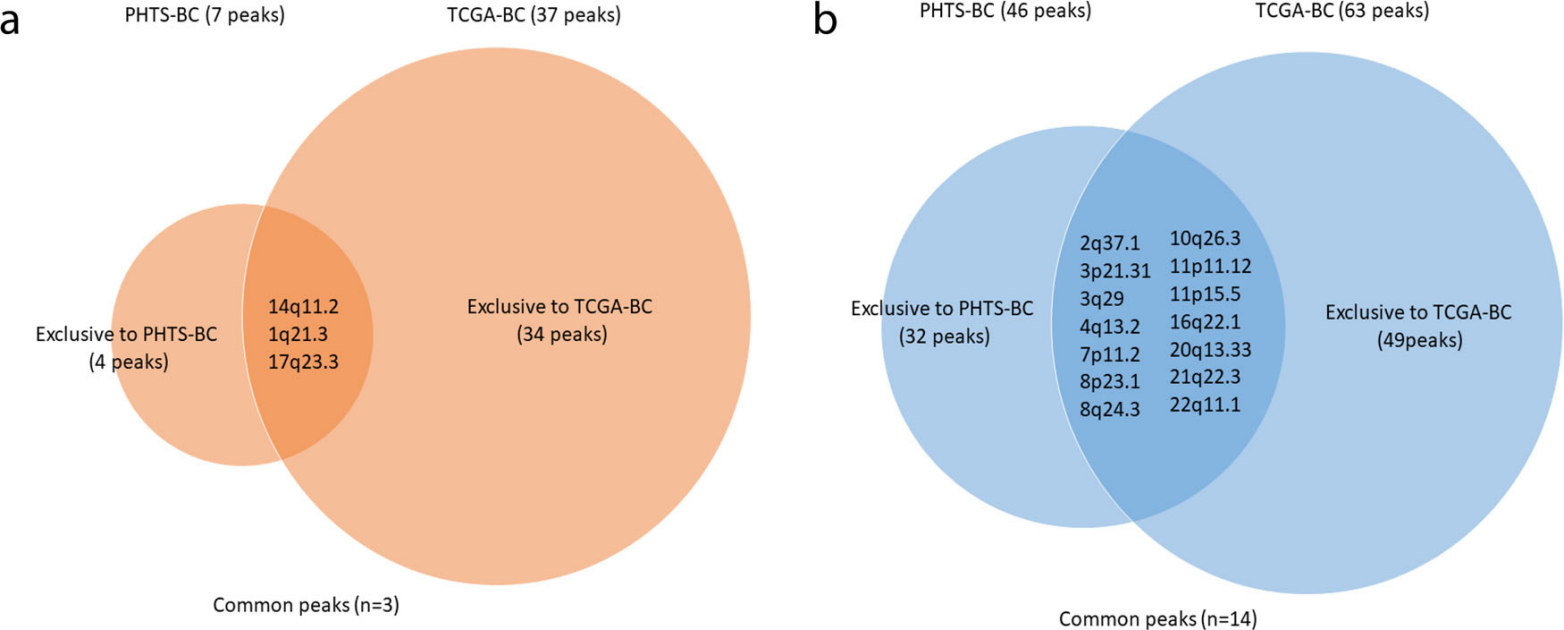
CNV peak comparison between PHTS-derived and TCGA BCs showing common and unique peaks. **a** Venn diagram showing three common amplification peaks between PHTS-derived BCs and TCGA BCs. **b** Venn diagram showing 14 common deletion peaks between PHTS-derived BCs and TCGA BCs.

Overall, there were three common peaks between PHTS-derived and TCGA BCs (Fig. 2a), while there were 14 common deletion peaks shared by the two groups (Fig. 2b).

### CNV and expression correlational analysis

In order to examine if any of the genes present within the identified CNV peaks are transcriptionally overexpressed for amplification peaks (7 peaks) or underexpressed for deletion peaks (46 peaks), we performed correlational studies between the height intensity of each peak and log2-fold expression changes (presence vs absence of the peak) for each gene in the peak regions. For significant peaks in PHTS-derived BCs, we found some genes are correlationally expressed and remained statistically significant after Bonferroni correction. For the amplification peak at 1q21.3, for instance, *ENSA* was correlationally over-expressed. There were no genes identified in the amplification peak at 14q11.2, but the closest gene by distance called *DAD1* was found to be correlationally overexpressed with this peak. For deletion peaks, the following genes were found to be correlationally underexpressed at the corresponding regions: *POLR2J4* at 7p13; *ANKRD20A1* at 9p21.11; *CNTNAP3B* at 9p11.2; *MUC5B* at 11p15.5; *CHEK2P2* at 15q11.1; *MYH1* at 17p13.1; and *OR7G1* at 19p13.2. (Supplementary Table 3). Among genes tested in the 6p22.2 region, *HIST1H2BI* was correlationally overexpressed by independent Fisher’s exact test (*p* = 0.0043). However, this finding did not remain statistically significant after Bonferroni correction (Supplementary Table 2).

### Difference in CNV between Tier-1 and Tier-2 PHTS-BCs

**Fig. 3:**
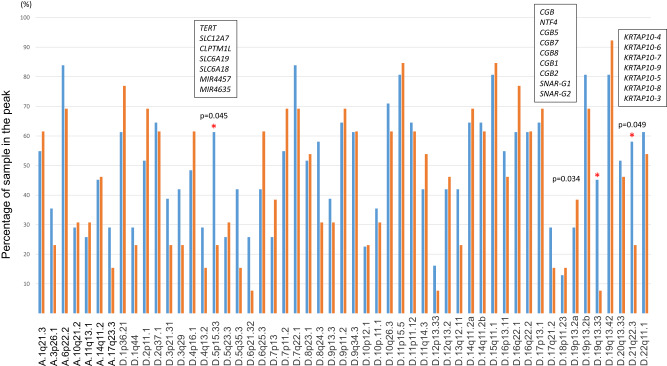
Comparison of CNV peak occurrences showed three chromosomal regions more frequently deleted in Tier-1 over Tier-2 derived BC samples. Bar graph showing amplification (A.) and deletion peaks (D.) at each significant CNV peak on the *X*-axis. The *Y*-axis represents the percentage of samples with statistically significant peaks present in each region. Blue bars represent Tier-1 derived BC samples, orange, Tier-2. Statistically significant different regions between Tier-1 and Tier-2 BCs are shown with a red asterisk along with the *p* value calculated by Fisher’s exact test. The genes contained in the regions with statistically significant tests are listed in the white boxes.

In our previous work, we studied two groups of tumors classified by the pathogenicity of the underlying germline *PTEN* variants. Tier-1 germline *PTEN* variants (*n* = 31) are classified as pathogenic or likely pathogenic, while Tier-2 germline *PTEN* variants (*n* = 13) as variants of unknown significance (VUS, *n* = 8) or likely benign (*n* = 5). To further characterize the differences between these two types of tumors, we examined which CNV peaks are more significantly amplified or deleted using Fisher’s exact test. Tier-1 BCs had three more significant deletion peaks compared to Tier-2 BCs, namely at 15p15.33, 19q13.33, and 21q22.3 (Fig. 3). None of the genes identified in these peaks have correlationally expressed transcripts.

### Differentially expressed genes between Tier-1 and Tier-2 PHTS-BCs

**Fig. 4:**
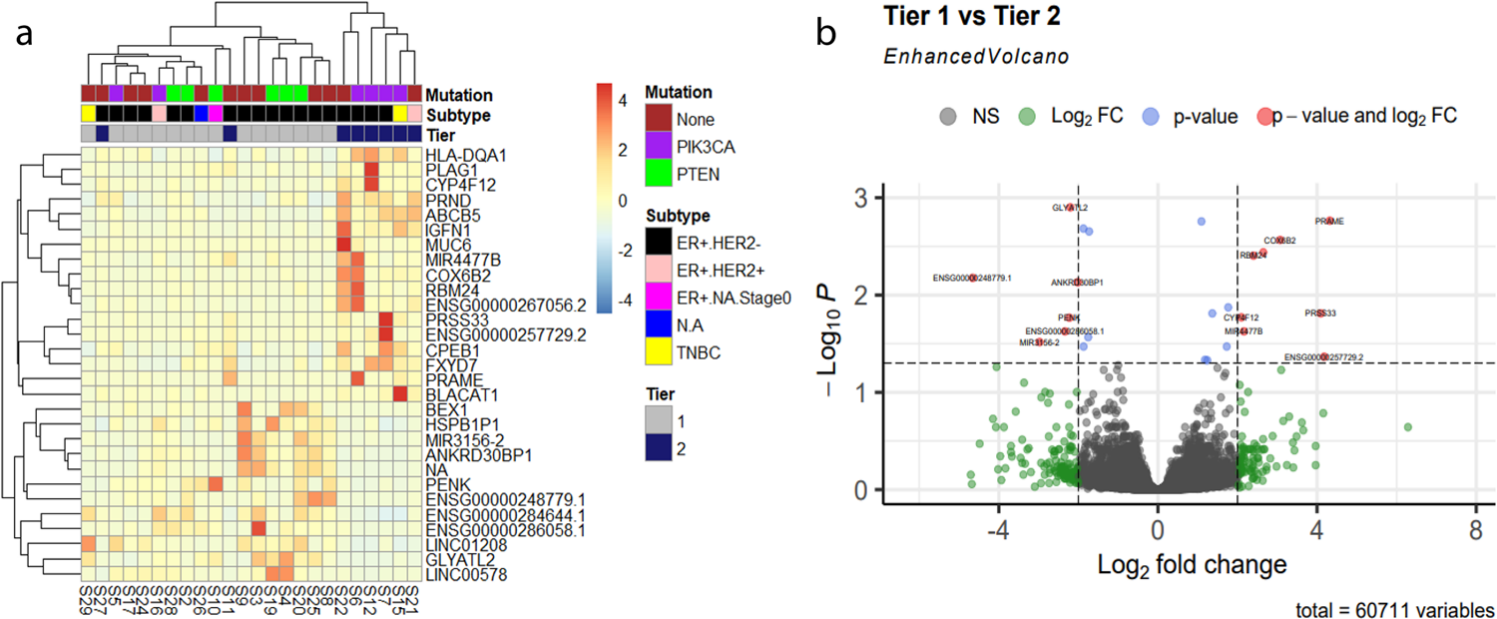
RNA-seq data showing two distinct groups in PHTS-derived BC samples, identifying alpha-tocopherol degradation as a significant biological pathway in Tier-1 PHTS-BC. **a** Heatmap of hierarchical clustering based on 28 differentially expressed (DE) genes, showing the PHTS-derived BC samples cluster into two groups, namely Tier-1 and Tier-2 (log2 fold change ± 1, *p* < 0.05). The *X*-axis lists the sample ID and right *Y*-axis shows the DE gene IDs included in this analysis. **b** Enhanced volcano plot of RNAseq transcriptome data showing differentially expressed genes in Tier-1 BCs compared to Tier-2 (log2 fold change ± 1, *p* < 0.05). The *x*-axis shows the magnitude of change (two fold change) and the *Y*-axis, statistically significance in −log10*P*.

We further examined the differences between Tier-1 and Tier-2 derived BCs at the transcriptome level by performing differential gene expression analysis. A hierarchical clustering heatmap showed Tier-1 and Tier-2 derived BCs clustered into two distinct patterns of differentially expressed genes (Fig. 4a). We identified a total of 18 differentially expressed genes with 10 overexpressed (*MUC6*, *PRAME*, *RP11_788H181*, *PRSS33*, *COX6B2*, *AC0053364*, *RBM24*, *IGFN1*, *mir-4477*, and *CYP4F12*) and 8 underexpressed (*RP11_53O192*, *BEX1*, *mir-3156*, *ANKRD30B*, *FAR2P1*, *PENK*, *GLYATL2*, and *ANKRD30BP1;* Fig.4b and Table 1). There were two Tier-2 derived BC samples which clustered among Tier-1 derived BC samples. Overall, we found no clear association between gene expression differences and BC subtypes or the presence of somatic *PTEN* or *PIK3CA* variants (Fig. 4b). The samples analyzed were also classified into intrinsic subtypes based on PAM50 to compare with clinical subtypes determined by immunohistochemistry (IHC) (Supplementary Table 5). The difference between Tier-1 and Tier-2 by intrinsic subtype was not statistically significant by chi-square test (*p* = 0.43).

**Table 1:** Top differentially expressed genes detected by DESeq2 (Tier-1 compared to Tier-2 BC).

Overexpressed genes	Fold changes
*MUC6*	5.065
*PRAME*	4.318
*RP11_788H181*	4.178
*PRSS33*	4.092
*COX6B2*	3.071
*AC0053364*	2.648
*RBM24*	2.402
*IGFN1*	2.178
*mir-4477*	2.161
*CYP4F12*	2.096

Underexpressed genes	Fold changes
*RP11_53O192*	−4.657
*BEX1*	−4.139
*mir-3156*	−2.975
*ANKRD30B*	−2.910
*FAR2P1*	−2.338
*PENK*	−2.227
*GLYATL2*	−2.204
*ANKRD30BP1*	−2.018

False discovery rate (FDR) threshold is 0.05 and log2 fold change threshold is ±1.

### Pathway analysis

We then examined which biological pathways are characteristic of Tier-1 BCs compared to Tier-2 BCs. Using the transcriptomic data from RNA sequencing as input, Ingenuity Pathway Analysis (IPA) revealed three enriched canonical pathways by Fisher’s exact test for Tier-1: (1) alpha-Tocopherol Degradation, (2) BEX2 Signaling Pathway, and (3) Oxidative Phosphorylation. After Benjamini-Hochberg correction, the first pathway, alpha-Tocopherol Degradation, remained statistically significant (*P* = 0.037).

### Immune cell population characterization and immunotherapy target gene abundance

**Fig. 5:**
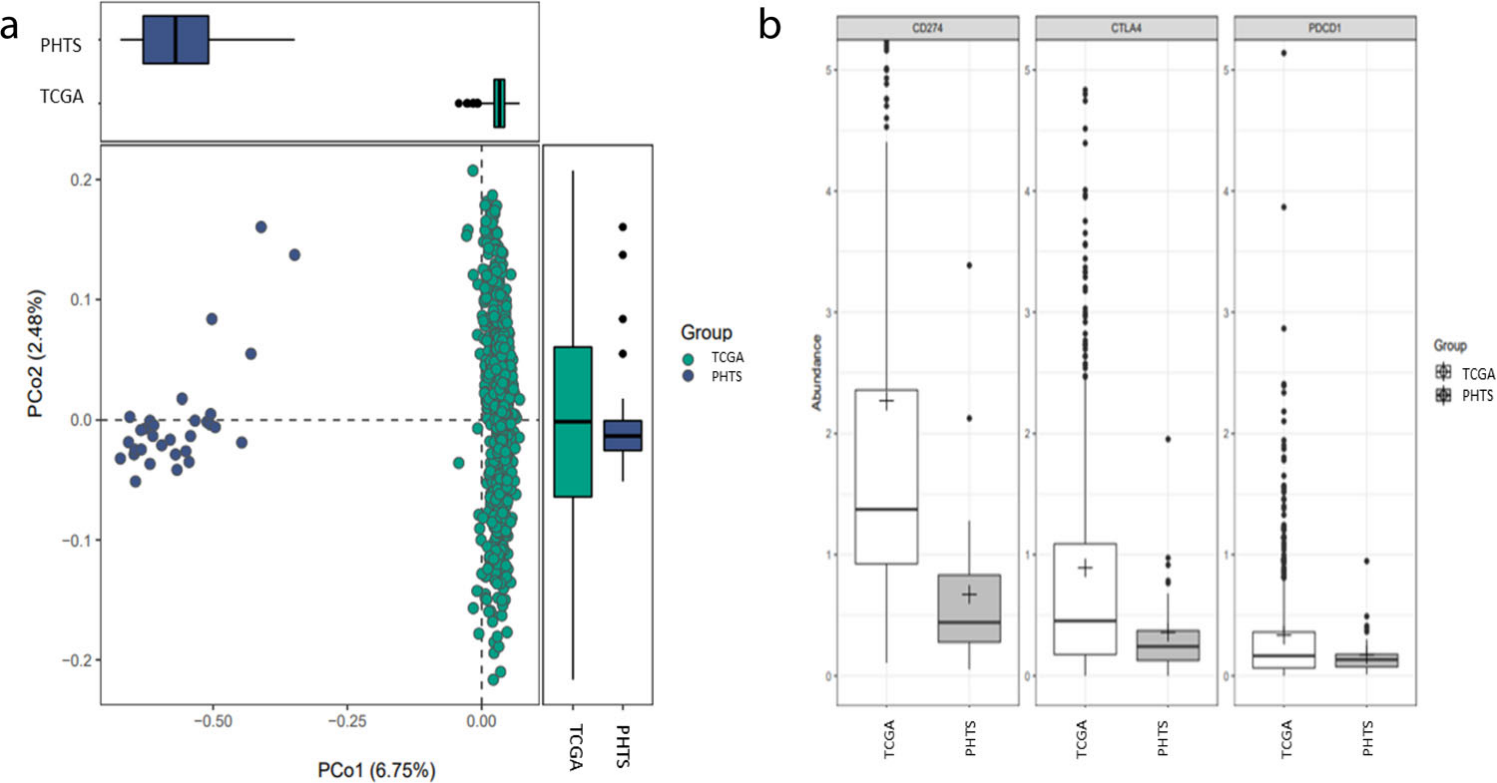
Immune cell population characterization and immunotherapy target gene abundance in PHTS-derived BCs vs sporadic BCs. **a** Beta-clustering revealed that PHTS-derived BC samples had distinct immune cell populations compared to sporadic BC samples from TCGA. Box plot showing beta-clustering based on fractions of each cell population, revealing that the PHTS BC group is distinct from the sporadic TCGA BC counterparts. **b** Gene abundance analysis revealed PHTS-derived BC samples had decreased abundance in three immunotherapy target genes. Box plot showing the gene abundance comparison between PHTS-derived BCs and TCGA BCs by Fisher’s exact test for PD-L1 (*CD274*), CTLA4, and PD-1 (*PDCD1*). TCGA BCs had significantly increased abundance of these genes (*CD274*, *p* = 0.014; *CTLA4*, *p* = 0.003; *PDCD1*, *p* = 0.001). For both (**a** and **b**): The bounds of each box represent the 25th and 75th percentiles (interquartile range [IQR]). The center line in each box represents the median. The top and bottom whiskers extend to the 5th and 95th percentiles, respectively. Solid circles are representing the possible outliers. Statistical significance between groups was tested using the Dunn method (*P* < 0.05). *P < 0.05, **P < 0.01, ***P < 0.001 and ns = non-significant.

In order to characterize patterns of immune cell populations infiltrating or surrounding breast carcinomas, we used CIBERSORT^9^ to impute immune cell compositions in PHTS-derived and TCGA-derived BCs. Beta-clustering based on fractions of each cell population showed that the PHTS BC group is distinct from the sporadic TCGA BC counterparts (Fig. 5a). We identified certain immune cell populations whose proportions are significantly increased in PHTS-derived BCs (*t*-test *p* < 0.05), namely naïve B cells, M0 macrophages, M2 macrophages, resting mast cells, monocytes, activated NK cells, and regulatory T cells (Supplementary Table 6, Supplementary Fig. 1). In contrast, the TCGA BCs had significantly greater predicted proportions of cell populations including dendritic cells (resting), eosinophils, M1 macrophages, mast cells, CD4 memory activated T cells, CD8 T cells, follicular helper T cells, and gamma delta T cells (Supplementary Table 6). Relatedly, we compared the gene abundance between PHTS-derived BCs and TCGA BCs for PD-L1 (*CD274*), *CTLA4*, and PD-1 (*PDCD1*). The TCGA BCs had significantly increased (Fisher’s exact test *p* < 0.05) abundance in these genes (*CD274*, *p* = 0.014; *CTLA4*, *p* = 0.003; *PDCD1*, *p* = 0.001; Fig. 5b).

## Discussion

In this study, we identified distinct somatic CNVs in PHTS-derived BCs compared to sporadic BCs. Overall, we observed notable heterogeneity across PHTS BC samples, which is consistent with the nature of BC biology in general. Our data point to several key findings, which help further characterize PHTS-derived BCs and offer insights into the biology of BCs arising in the setting of germline *PTEN* variants.

Our data revealed somatic CNVs in PHTS-BC which are distinct from those in sporadic BCs from TCGA. The most significant amplification peak was at 6p22.2, which was not observed in TCGA. This peak contains several histone-related genes, including *HIST1H2BI*, which was found to be correlationally expressed with the copy number change. The lack of statistical significance in correlational expression of the other histone genes may be due to the limited sample size. Furthermore, although not statistically significant, there was a higher proportion of somatic *PTEN* variants in the samples which exhibited significant amplification at 6p22.2. The PTEN protein is known to interact with histone H1 to maintain chromatin organization and integrity^10^. Importantly, we previously demonstrated that the tumor mutational burden is increased in PHTS-derived BCs compared to sporadic BCs, which supports genomic instability as an important component of BC biology in PHTS^5^. When PTEN dysfunction negatively affects chromatin stability, this leads to dysregulated gene expression^10^. We therefore speculate that the significant 6p22.2 amplification peak may represent a feedback loop to compensate for the compromised genome integrity and increased instability. In such a case where PTEN is severely dysfunctional, leading to genomic instability, therapeutic agents targeting DNA damage may be useful, including DNA intercalating agents such as doxorubicin and poly(ADP-ribose) polymerase (PARP) inhibitors^11^.

We found that for the amplification peak at 1q21.3, the cis-gene alpha-endosulfine gene (*ENSA*) was found to be correlationally over-expressed in PHTS relative to TCGA BCs. This peak was also present in BC samples from TCGA and is known to be a recurrent amplification in BC. *ENSA* has been found to be highly expressed in triple negative breast cancer (TNBC) and associated with poor survival in this group^12^. Upregulation of *ENSA* has been shown to promote tumor growth by regulating cholesterol biosynthesis^12^. This may be one of the common biological mechanisms for carcinogenesis shared by PHTS-derived and sporadic BCs.

For deletion peaks, it is unknown how altered gene expression due to copy number deletion may contribute to carcinogenesis in BC. RNA Polymerase II Subunit J4, Pseudogene (*POLR2J4*) at 7p13 and Olfactory Receptor Family 7 Subfamily G Member 1 (*OR7G1*) at 19p13.2 are reported to be associated with non-breast cancers^13,14^. Contactin Associated Protein Family Member 3B (*CNTNAP3B*) at 9p11.2 has been reported to be overexpressed in atypical hyperplasia of the breast^15^. Mucin 5B, Oligomeric Mucus/Gel-Forming (*MUC5B*) at 11p15.5 was also found to be correlationally expressed with the peak at 11p15.5. This peak was also identified in the TCGA group. Previous studies have shown *MUC5B* expression was increased in BCs compared to normal breast epithelium^16^, and that *MUC5B* expression is associated with aggressive behavior of BC cell lines^17^. There is currently little evidence in the literature describing checkpoint kinase 2 pseudogene 2 (*CHEK2P2*) at 15q11.1, Myosin Heavy Chain 1 (*MYH1*) at 17p13.1 *or* Ankryn Repeat Domain 20 Family Member A1 (*ANKRD20A1*) at 9q21.11 as significant genes in BC. Whether and how these copy number deletions and gene expression differences play a role in breast carcinogenesis in PHTS warrants further investigation.

There are two biologically distinct groups of PHTS-derived BCs based on the pathogenicity of the underlining germline *PTEN* variants: (1) Tier-1 variants are classified as pathogenic or likely pathogenic; and (2) Tier-2 variants, as variants of unknown significance (VUS) or likely benign^5^. Our previous exome sequencing data revealed that Tier-1 and Tier-2 derived BCs are different at the genomic level. This finding was further supported by transcriptomic analysis data, where Tier-1 and Tier-2 BCs clustered separately. Some genes overexpressed in Tier-1 BCs relative to Tier-2 BCs may contribute to BC tumorigenesis and progression. For instance, some members of the mucin protein family, have been shown to be highly expressed in mucinous BC and associated with negative estrogen receptor (ER) status^18^. Expression of PReferentially expressed Antigen of Melanoma (*PRAME*) was previously shown to correlate with poorer clinical prognosis, including higher rates of distant metastases and decreased overall survival in BC^19^. Additionally, the PRAME protein has been investigated as a potential immunotherapy target^20,21^. Serine Protease 33 (*PRSS33*) and Cytochrome C oxidase subunit 6B2 (*COX6B2*) are not well-characterized in BC but their expression is associated with other types of cancer^22,23^. Similarly, other overexpressed genes including RNA binding motif protein 24 (*RBM24*), Immunoglobulin-like and fibronectin type III domain containing 1 (*IGFN1*), and Cytochrome P450 family 4 subfamily F member 12 (*CYP4F12*) may have biological contributions to tumorigenesis in Tier-1 PHTS BC but their exact roles are unclear^24–28^. Additional investigation of their association with BC is warranted.

The contribution of Tier-1 underexpressed genes to tumorigenesis and disease progression is even less clear. Little is known about the molecular functions of brain expressed X-linked 1 (*BEX1*) and its exact role in tumorigenesis is still under debate^29,30^. Proenkephalin (*PENK*) is one of the genes encoding for endogenous opioid precursors^31^. Interestingly, downregulation of *PENK* is reported to be associated with defects in cell motility and abnormal adhesion in brain metastasis from BC^32^. Glycine-N-acyltransferase like 2 (*GLYATL2*) is a glycine conjugating enzyme with functions implicated in barrier function and immune response^33^. Very little is known about any association between ankyrin repeat domain 30B (*ANKRD30B*) and any type of cancer.

Although some of the identified differentially expressed genes have been implicated in BC development and progression, we do not think one or just a few genes drive tumorigenesis in PHTS-derived BCs. Thus, we performed pathway analysis, which revealed ᾳ-tocopherol degradation to be a significantly impacted canonical pathway in Tier-1 vs Tier-2 BCs. Also known as vitamin E, ᾳ-tocophenol is an antioxidant^34^, and an animal and cell-based study has shown that vitamin E may increase PTEN and p53 levels in the rat prostate^35^. Furthermore, in a subtype of Cowden syndrome with no germline *PTEN* mutations but with germline Succinate dehydrogenase (*SDHx)* variants, vitamin E appears to protect from oxidative stress and potentially suppresses tumorigenesis^36^. We hypothesize that vitamin E plays an important role in suppressing the development of cancer in cells with dysfunctional PTEN-related pathways. Being a key pathway in Tier-1 BCs, vitamin E degradation may explain the more penetrant nature of pathogenic germline *PTEN* variants including within the Tier-1 BCs due to enhanced elimination of vitamin E, which is supposed to protect cells from carcinogenic oxidative damage. This hypothesis is worth experimentally testing, including in relevant *Pten* animal models.

The immune landscape infiltrating or surrounding the breast carcinomas appears distinct between PHTS and TCGA. Overall, beta-clustering revealed these two groups to be significantly different from one another in cell composition, with certain immune cell populations predicted to be significantly increased in proportion either in PHTS or TCGA BCs. More specifically, immune cell populations which are either inactive or suppressive (naïve B cells, M0 macrophages, M2 macrophages, resting mast cells, monocytes, and regulatory T cells) are increased in PHTS BCs. Furthermore, genes encoding immune checkpoint pathways, including PD-L1 (*CD274*), CTLA4, and PD-1 (*PDCD1*), were found less abundant in PHTS-derived BCs, suggesting that the PHTS-derived BCs may be less responsive to immune checkpoint inhibitors.

Consistent with our findings in PHTS BC, previous studies focusing on sporadic BCs also showed that PTEN deficiency in tumors is associated with an immunosuppressive tumor microenvironment (TME) and resistance to immune checkpoint blockade^37,38^. Mechanistically, intrinsic PTEN deficiency in tumor cells stimulates the activation of phosphoinositide 3-kinase (PI3K) signaling and the secretion of VEGF, which lead to the recruitment of immunosuppressive immune cells, abnormal angiogenesis, and resistance to T cell-mediated killing^38^. In contrast, distinct from sporadic cancers, PTEN deficiency in PHTS BCs occurs not only in tumor cells, but also in the non-malignant normal cells (germline effect), including immune and stromal cells, which could influence the differentiation, expansion, activation, trafficking, and phenotypes of immune and stromal cells in the TME as well. For example, a previous study has found that genetic depletion of PTEN enhances NK cell cytolytic function against malignant cells, which is consistent with our data that increased proportion of activated NK cells was found in PHTS BCs^39^. Accordingly, to design strategies for immunotherapy in PHTS BCs, the influences of the PTEN pathway in both tumor and non-tumor (especially immune) compartments need to be considered. Notably, mutational signatures were also found to be associated with phenotypes of the TME and responsiveness to immunotherapy. For example, contrary to smoking associated signatures that show better response to immunotherapy, age-related mutational signature was found negatively associated with immune activity, survival outcomes, and the response to immunotherapy in triple-negative BC, melanoma, and/or NSCLC^40,41^. Our finding that PHTS BCs contain enriched age-related mutational signature provides another potential linkage between PTEN deficiency and defective anti-tumor immune responses in PHTS BCs^5^.

In conclusion, this study reveals key genomic and transcriptomic alterations in PHTS-derived BCs which are distinct from those of the sporadic BC group from TCGA. We further revealed a potential key pathway associated with BC biology in PHTS, especially in the setting of pathogenic germline *PTEN* mutations. The alterations we identified enable hypothesis-driven studies to further characterize downstream functional effects contributing to BC carcinogenesis in PHTS. PHTS will only rise in incidence as clinical genetic testing becomes more widely accessible in the clinic. Currently, there are no PHTS-specific treatment strategies for any type of PHTS component cancers, including BC. More extensive studies both at the clinical, translational and basic science levels are warranted to develop PTEN-targeted and personalized treatments, and perhaps preventatives, to effectively manage PHTS-derived cancers.

## Methods

### Research participants

Approved by the Cleveland Clinic’s Institutional Review Board (IRB), written informed consents were obtained from all individuals enrolled under the study protocol. Among 6934 research participants prospectively accrued from September 1, 2005 to September 10, 2020, we identified 3066 female participants with a personal history of breast cancer (BC). Of these, 130 had germline PTEN variants. We then identified 44 women with appropriate consents for acquisition of biospecimens and whose tissues representing BC were available for sequencing.

Original formalin-fixed paraffin-embedded (FFPE) samples representing primary breast carcinoma were obtained from healthcare institutions where the pathology specimens were originally collected. DNA was extracted from the FFPE blocks using QIAamp® DNA FFPE Tissue kit (Qiagen, Venlo, Netherlands). Matched blood-derived DNA originating from lymphoblastoid cell lines from the subjects were obtained from the Genomic Medicine Biorepository at the Lerner Research Institute of the Cleveland Clinic (Cleveland, OH, USA). Baseline patient characteristics including histologic subtypes, BC-specific tumor markers, age of diagnosis, staging, grade, germline *PTEN* variants and their classifications, were extracted from the Cleveland Clinic Genomic Medicine Institute’s relational database and as previously described^5^.

### DNA extraction

DNA was extracted from the FFPE samples using QIAamp® DNA FFPE Tissue kit (Qiagen, Maryland, USA). Briefly, tissues from FFPE blocks were deparaffinated with xylene and crude DNA was precipitated with 100% ethanol. Following complete proteolysis of the samples with Proteinase K at 56 degrees Celsius, DNA was extracted and purified using the column method according to the manufacturer’s protocol with slight reagent volume modifications. For matched germline samples, we obtained blood-derived genomic DNA originating from whole blood from the PHTS individuals from the Genomic Medicine Biorepository of the Genomic Medicine Institute at the Cleveland Clinic (Cleveland, OH, USA) following standard procedures.

### Processing of extracted DNA samples

Samples with sufficient DNA yields and quality were subjected to exome sequencing. DNA concentration was measured with the Qubit™ Fluorometer dsDNA HS (High Sensitivity) Assay kit (Thermo Fisher Scientific, Waltham, Massachusetts, USA). While the ideal DNA concentration for sequencing library preparation was considered to be 30–40 ng/µL, the range of DNA concentrations of submitted samples was 9.6–68.4 ng/µL and 19.0–98.4 ng/µL, and the range of sample volumes submitted was 30–45 µL and 30–60 µL for tumor and normal samples, respectively.

### Exome sequencing

Next generation sequencing (NGS) was performed on the tumor-blood DNA pairs using the Illumina HiSeq platform at the Broad Institute of MIT and Harvard University. The raw data were quality controlled, aligned and sorted by the computational pipeline at the Broad Institute to generate binary alignment map (BAM) files for tumor and blood samples separately. The Broad Institute created libraries from the submitted DNA samples and used the Illumina HiSeq platform to generate NGS data. Of the 44 tumor-normal samples, 28 were processed with the Illumina Somatic Exome protocol and the remaining 16 with the TWIST Somatic Exome protocol (pair-end sequencing with read length range of 67–140 bp). The Illumina Somatic Exome protocol had target depths of 20× and 50× for the normal and tumor samples, respectively. For the TWIST Somatic Exome protocol, the target depth was ×100 for both normal and tumor samples. The raw data were quality controlled, aligned and sorted through a standard NGS pipeline at the Broad Institute. Reads were aligned to the reference human genome GRCh37/hg19 using the BWA-ALN aligner (version 0.5.9)^42^. Local realignment, duplicate removal and base quality score recalibration were performed using the Genome Analysis Toolkit and Picard per the Broad Institute standard protocol^43^. The processed sequencing data, derived from both tumor and blood samples, were delivered as binary alignment map (BAM) files.

### Sporadic breast cancer cohort

The control cohort data were derived from The Cancer Genome Atlas (TCGA) breast cancer dataset from the Genomic Data Commons (GDC). BC cases with available exome sequencing data were selected. Cases with germline mutations in known cancer susceptibility genes were identified based on previously published data^44^ and excluded. Pertinent clinical information of the selected cases was obtained from Nationwide Children’s Hospital dataset, which is publicly available from the GDC portal (universally unique identifier [UUID] 8162d394-8b64-4da2-9f5b-d164c54b9608).The original input files (BAMs) of tumor and matched normal samples, aligned to reference human genome GRCh38/hg38, were downloaded from the GDC archive website for bioinformatics analyses.

### Copy number variation analysis

With the WES data from 44 PHTS-derived BC samples and 558 sporadic BC samples from TCGA as input, the segmentation and raw copy number data were obtained using FACETS (version 0.5.6), an open-source tool to analyze allele-specific copy number variations^45^. The critical value (cval) was specified at 50 to create an input for gistic2 (version 6.15.28)^46^, which identifies significantly recurrent copy number alterations in the somatic genome. We used the following setting: a confidence interval of 95%, *q* value of 0.01, amplification threshold of 0.3, and deletion threshold of −0.3. For other parameters, we used the default setting specified by gistic2. We applied the same CNV algorithm to the raw TCGA sporadic BC dataset to make a head-to-head comparison with our PHTS series data.

### Patients and RNA extraction

FFPE tissue samples were available from a subset of the PHTS BC series (*n* = 29). RNA was extracted from the available FFPE blocks using AllPrep® DNA/RNA FFPE kit or RNeasy FFPE kit (Qiagen). RNA concentration was measured with the Qubit Fluorometer dsDNA HS (High Sensitivity) Assay kit (Thermo Fisher Scientific, Waltham, MA, USA). While the ideal RNA concentration for sequencing library preparation was considered to be 30–40 ng/mL, the range of RNA concentrations of submitted samples was 57.6–540 ng/mL. The range of 280/260 ratio was 1.81 to 2.07. The extracted RNA was sent to the Genomics Core of the Department of Genetics and Genome Sciences at Case Western Reserve University (Cleveland, OH, USA) for library construction. The constructed RNA libraries were then sent to the Genomics Core at the Cleveland Clinic Lerner Research Institute (Cleveland, OH, USA) for RNA sequencing.

### RNA-seq library preparation and sequencing

The SMARTer Stranded Total RNA-Seq Kit v2 Pico Input Mammalian from Takara Bio USA (protocol 050619) was used to prepare RNA-Seq libraries. The total RNA input was adjusted to 100 ng in 8 ul of nuclease-free water. Since FFPE samples intrinsically have highly degraded RNA, cDNA synthesis was performed without fragmentation. Subsequent PCR steps utilized the indexes from the SMARTer RNA Unique Dual Index Kit – 24U (634451). Ribosomal cDNA was depleted, and the final amplification included 13 cycles of PCR. Samples were purified with AMPure beads and eluted in 18 µl of 5 mM Tris Buffer. Final QC included running samples (diluted 1:1 in water) on the HSD1000 tape on the Agilent TapeStation and obtaining a Qubit reading (Thermo Fisher Scientific).

The constructed libraries were sequenced on an Illumina NovaSeq 6000 using an S2 flow cell, where dual-indexed paired-end 151 bp sequencing was accomplished. Sequencing data were demultiplexed using *bcl2fastq* and FastQC reports were generated to evaluate the sequence quality of each sample.

### Differentially expressed gene analysis

Based on the FastQC report of the original FASTQ files generated by RNA sequencing, we noted that the first three base pairs in reverse reads (R2) consistently had low quality scores at the 5′ end. Thus, the first three base pairs at the 5′ end in R2 FASTQ files were clipped using trimmomatic (version 0.39)^47^. Adaptor and ribosomal sequences were trimmed off using BBmap (version 37.96)^48^. The optimized FASTQ files were then aligned to hg38 using STAR (version 2.7.8)^49^.

FastQC reports were again obtained on STAR aligned FASTQ files. Five out of 29 samples had less than 50% uniquely mapped reads, and were excluded from differentially expressed gene analyses. We analyzed the 24 samples quality control using DESeq2 (version 1.34.0) to identify differentially expressed genes with statistical significance^50^, defined as a false discovery rate (FDR) < 0.05 and a log2-fold change > +/− 1 (>2 for overexpression and less than −2 for underexpression).

The hierarchical clustering heatmap was created using pheatmap (version 1.0.12)^51^ and the volcano plot was created using EnhancedVolcano (version 1.12.0)^52^, using R (version 4.1.2).

### Intrinsic subtype determination

We used genefu, an R package, to classify 26 PHTS-BC derived RNA samples into the basal, Her2, luminal A, luminal B, and normal-like intrinsic subtypes based on PAM50. Genefu is available at its Bioconductor site (http://www.bioconductor.org/packages/release/bioc/html/genefu.html).

### CNV and transcriptome correlational analysis

We performed correlational studies to examine which cis-genes are correlationally expressed with the chromosomal peaks detected by gistic2. For each sample, the log2 fold change raw values from DESeq2 were tested for the actual copy change values from gistic2. Pearson correlation analysis^53^ was used for genes with normally distributed log2 fold changes, and Spearman correlation^54^ for those with non-normal distributions. Normalization test was performed using D’Agostino-Pearson omnibus normality test, Anderson-Darling test, Shapiro–Wilk normality test and Kolmogrov-Smirnov normality test with the default setting with alpha of 0.05 on GraphPad Prism version 9.0 (GraphPad Software, San Diego, CA, USA). Bonferroni correction was performed to identify statistically significant genes associated with the peak regions containing multiple genes.

### Breast cancer-associated genes

For targeted analysis, we aggregated lists of genes associated with BC^5^. The selected genes were chosen from the TCGA BC publication^55^, NCCN Genetic/Familial High-Risk Assessment: Breast and Ovarian guidelines (version 1.2022-August 11, 2021), 22 previously reported gold standard (GS) genes for BC^56^, preliminary BC susceptibility genes and targetable BC-associated genes from the literature^56–75^. A total of 84 BC-associated genes were included in the final list (Supplementary Table 1).

### Pathway analysis

Output from DESeq2, including HUGO Gene Nomenclature Committee (HGNC) gene ID, log2 fold changes and adjusted *p* values, was uploaded into the Ingenuity Pathway Analysis (IPA) software (QIAGEN, Venlo, Netherlands). The data were then subjected to functional annotations and canonical pathway analyses. The IPA’s Core Analysis workflow was performed using default parameters. For Benjamini-Hochberg (B-H) correction, the score cut off (A-log or B-H *p* value) of >1.3 was used.

### CIBERSORT and diversity analyses

The TCGA Breast Invasive Carcinoma (BRCA) RNA-Seq dataset was downloaded using TCGAbiolinks package^76^. Data retrieval was performed by the three main TCGAbiolinks functions: GDCquery, GDCdownload and GDCprepare. The raw feature count matrix was converted to transcripts per million (TPM) and merged with PHTS data. The merged TPM matrix was processed for differential abundance analysis using the random-forest algorithm, implemented in the DAtest package (https://github.com/Russel88/DAtest/wiki/usage#typical-workflow). Briefly, the performance of differential abundance methods was compared with False Discovery Rate (FDR), Area Under the (Receiver Operator) Curve (AUC), Empirical power (Power), and False Positive Rate (FPR). Based on the DAtest’s benchmarking, we selected random forest as the method of choice to perform differential abundance analysis. We assessed the statistical significance (*P* < 0.05) throughout, and whenever necessary, we adjusted *p* values for multiple comparisons according to the Benjamini-Hochberg method to control false discovery rate while performing multiple testing on gene abundance according to sample categories. We used CIBERSORT^9^ to perform RNA-Seq deconvolution analysis and estimate immune cell fractions in our bulk RNA-Seq data. We used SVASeq to perform the batch correction^77^.

### Statistical analysis

Statistical analyses were performed with GraphPad Prism version 9.0 (GraphPad Software, San Diego, CA, USA), except for statistical analyses incorporated in maftools (version 2.10.0)^78^. *P* values < 0.05 were considered statistically significant unless otherwise stated. Two-sided tests were used unless otherwise stated.

### Sample size estimation

We performed sample size calculations to determine the minimum number of cases we need to be powered to identify statistically significant genomic differences between the PHTS and TCGA sporadic BC groups. In order to detect characteristic differences at the variant level, we used the two proportions derived from the somatic *PTEN* mutation rate in the preliminary PHTS group with 29 samples (21.0%) and that of sporadic luminal subtypes in the literature (4.0%)^55,79^. We estimated that 30 samples from PHTS and 250 samples from TCGA should be sufficient to achieve a power of 81.0% with an alpha of 0.05 (two-sided) to detect a significant difference. RNA was extracted from samples with sufficient tissue materials.

### Reporting summary

Further information on research design is available in the Nature Research Reporting Summary linked to this article.

## Supplementary information

Supplemental Information

Supplementary Table 4

Supplementary Table 5

Reporting Summary

## Data Availability

Our institutional IRB and Legal Department do not permit clinical information or genomics data reposited in a publicly accessible database at this time (by policy). Thus, requests for such data relevant to this paper should be made to the corresponding author Prof. Eng (engc@ccf.org). Thereafter, the Legal Department will ask for a materials transfer agreement and data sharing agreement to be executed.

## Code availability

Code for data analysis used in this study is available at https://github.com/brew-t2/BC_CNV_analysis. All the bioinformatics tools used are publicly available and referenced accordingly.
