# Supplementary material for: Integrating somatic CNV and gene expression in breast cancers from women with PTEN hamartoma tumor syndrome
Source: NPJ Genom Med. 2023 Jul 5;8:14. doi: 10.1038/s41525-023-00361-0 (PMC10322985; doi:10.1038/s41525-023-00361-0)
Supplement: Supplementary file 1 — Supplemental Information [file 41525_2023_361_MOESM1_ESM.pdf]

Supplementary Figures and Tables

Integrating Somatic CNV and Gene Expression in Breast Cancers from Women with *PTEN* Hamartoma Tumor Syndrome

Takae Brewer, Lamis Yehia, Peter Bazeley, Charis Eng

Supplementary Fig. 1 CIBERSORT reveals heterogeneity of immune cell populations in PHTS-derived BC samples

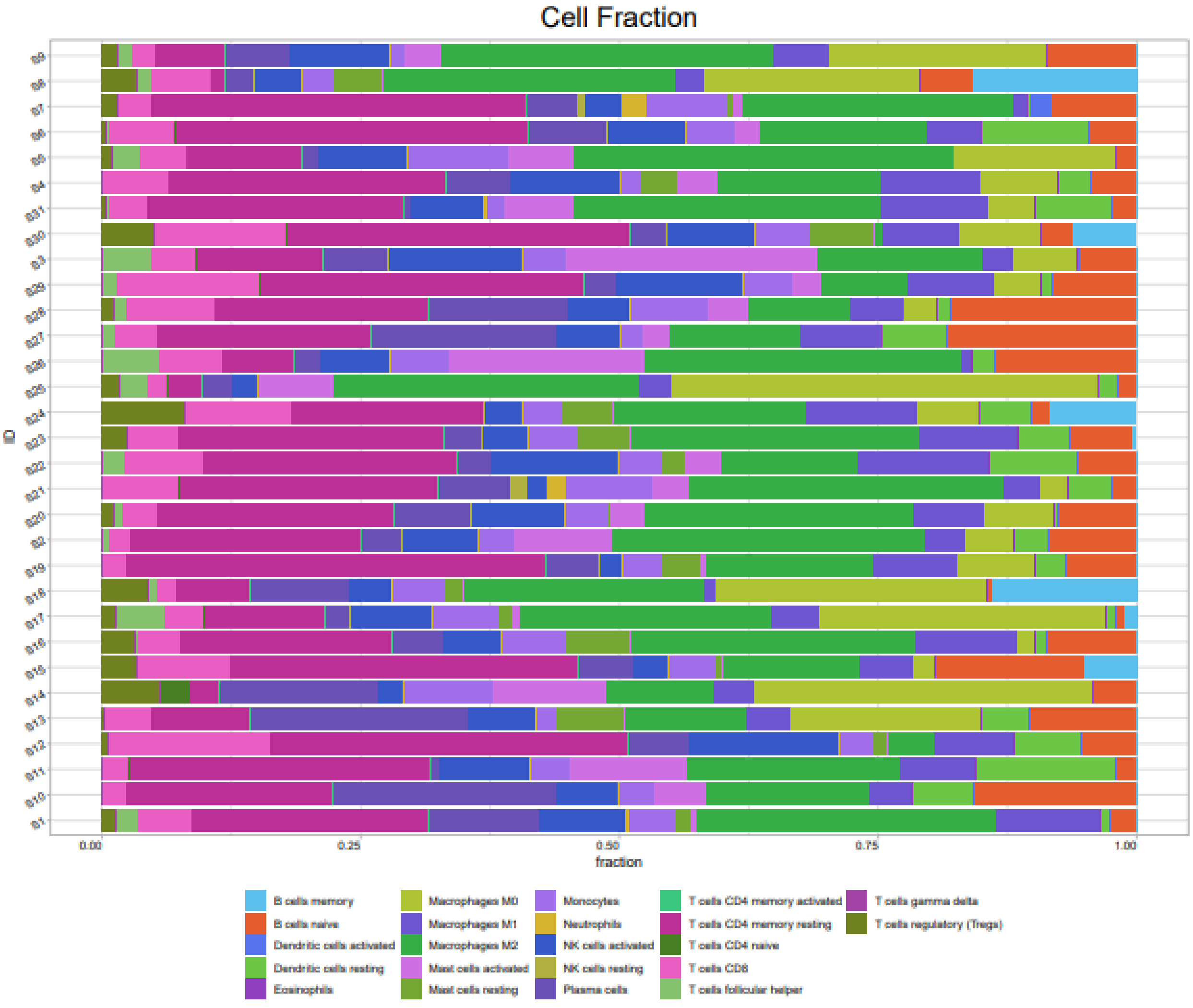

CIBERSORT plots showing cell compositions in PHTS-derived BC samples. X-axis shows the estimated percentage of each cell population. Each row represents a sample with the corresponding sample ID.

Supplementary Tables

Supplementary Table 1. Aggregated breast cancer (BC) associated genes

Gene Name	Chrom	Coordinates (GRCh37/hg19)	
		Start	*End*
ABRAXAS1	4	84380669	84406253
AFF2	X	147582136	148082193
AKT1	14	105235685	105262085
AKT3	1	243651534	244006584
APC	5	112043194	112181936
AR	X	66763862	66950461
ARID1A	1	27022505	27108595
ATM	11	108093793	108239829
BARD1	2	215590369	215674407
BRAF	7	140413127	140624729
BRCA1	17	41196311	41277381
BRCA2	13	32889644	32974405
BRIP1	17	59756499	59940889
CASP8	2	202098165	202152434
CBFB	16	67063051	67134961
CCND1	11	69455923	69469242
CCND2	12	4382927	4414519
CCND3	6	41902670	42016632
CCNE1	19	30302897	30315219
CD274	9	5450541	5470554
CDH1	16	68771194	68869440
CDK4	12	58141509	58146093
CDK6	7	92234234	92465887
CDKN1B	12	12870301	12875303
CDKN2A	9	21967750	21995323
CDKN2B	9	22002901	22009312
CHEK2	22	29083730	29137822
CTCF	16	67596428	67673080
EGFR	7	55086709	55279321
ERBB2	17	37844346	37884911
ERBB3	12	56473948	56497289
ESR1	6	151977806	152424409
ESR2	14	64693424	64805331
FANCM	14	45605132	45670093

FBXW7	4	153241695	153457244
FGFR1	8	38268660	38326153
FGFR2	10	123237843	123357972
FOXA1	14	38058756	38064454
GATA3	10	8096650	8117161
IGF1	12	102789651	102874341
JAK2	9	4984389	5129948
KLLN	10	89618914	89623290
KMT2A	11	118307206	118397547
KMT2C	7	151832009	152133088
KRAS	12	25358179	25403863
MAP2K1	15	66679249	66783882
MAP2K4	17	11924193	12047145
MAP3K1	5	56111375	56191979
MET	7	116312249	116438431
MRE11	11	94148735	94227010
MUTYH	1	45794913	45806112
MYB	6	135502445	135540310
NBN	8	90945558	90996895
NCOR1	17	15932470	16118848
NF1	17	29421944	29704695
NOTCH1	9	139388884	139440500
NOTCH4	6	3505440	3534689
NTHL1	16	2089820	2097835
PALB2	16	23614485	23652631
PGR	11	100900354	101000544
PIK3CA	3	178866144	178957881
PIK3CB	3	138371539	138553770
PIK3R1	5	67511583	67597649
PTEN	10	89623381	89731687
PTPN22	1	114356432	114414381
PTPRD	9	8314245	10613002
RAD50	5	131892668	131982041
RAD51C	17	56769962	56812972
RAD51D	17	33419239	33446879
RB1	13	48877886	49056026
RECQL	12	21621843	21654569
RINT1	7	105172647	105208124
RPTOR	17	78518637	78940168
RUNX1	21	36160097	36421599
SDHB	1	17345216	17380527
SDHD	11	111957596	111966518
SEC23B	20	18488191	18542059

SF3B1	2	198283519	198299817
STK11	19	1205776	1228430
TBL1XR1	3	176737131	176915270
TBX3	12	115108059	115121980
TP53	17	7571738	7590808
WWP1	8	87354775	87480732
XRCC2	7	152341860	152373226

Supplementary Table 2. Amplification peaks detected by gistic2 in PHTS-derived BCs

	Amplification peak ***(no. of genes in the peak region)***	***Genes in the region*** ***(unadjusted p-value)***
1	1q21.3 (n=4)	*ENSA** (p=0.012)
2	3p26.1	-
3	6p22.2 (n=11)	*HIST1H2BI** (p=0.004)
4	10q21.2 (n=3)	*ARID5B* (p=0.024)
5	11q13.1	-
6	14q11.2 (n=1)	*[DAD1]** (p=0.019)
7	17q23.3 (n=24)	-

The number of genes found in each peak region is shown in parenthesis in the first column. Genes in regions whose expression were statistically correlated with the peak intensities are shown in the second column. The p-values shown in column 2 are unadjusted. After Bonferroni correction, based on the number of genes tested within each region, the genes indicated with an asterisk remained statistically significant.

Supplementary Table 3. Deletion peaks detected by gistic2 in PHTS-derived BCs

	Deletion peak ***(no. of genes in the peak region)***	***Genes in the region*** ***(unadjusted p-value)***
1	1p36.21 (n=19)	*PRAMEF20* (p=0.046)
2	1q44 (n=20)	*OR2M4* (p=0.041), *OR2M3* (p=0.0039), *OR2T6* (p=0.022)
3	2p11.1	-
4	2q37.1	-
5	3p21.31 (n=5)	*MIR-711* (n=0.029)
6	3q29	-
7	4p16.1	-
8	4q13.2	-
9	5p15.33	-
10	5q23.3	-
11	5q35.3 (n=3)	*SCGB3A1* (p=0.034)
12	6p21.32	-
13	6q25.3	-
14	7p13 (n=3)	*POLR2J4** (p=0.013)
15	7p11.2	-
16	7q22.1	-
17	8p23.1	-
18	8q24.3 (n=4)	*LY6E* (n=0.016)
19	9p13.3	-
20	9p11.2 (n=35)	*ANKRD20A1** (p=0.0006), *CNTNAP3B** (n=0.0011), *SPATA31A1* (n=0.0015)
21	9q34.3	-
22	10p12.1 (n=5)	*GAD2* (p=0.018), *FAM238B* (p=0.018)
23	10p11.1	-

24	10q26.3 (n=9)	*KND1* (p=0.0055)
25	11p15.5 (n=3)	*MUC5B** (p=0.0007)
26	11p11.12 (n=59)	*ORSM11* (p=0.018), *OR5J2* (p=0.040)
27	11q14.3 (n=16)	*FOLH1B* (p=0.012)
28	12p13.33	-
29	12q13.2 (n=16)	*OR10P1* (p=0.021)
30	13q12.11 (n=6)	*TPTE2* (p=0.025), *ANKRD26P3* (p=0.032)
31	14q11.2 (peak A)	-
32	14q11.2 (peak B)	-
33	15q11.1 (n=1)	*CHEK2P2** (p=0.035)
34	16p13.11	-
35	16q22.1 (n=5)	ARRS1 (p=0.011)
36	16q22.2	-
37	17p13.1 (n=7)	*MYH1** (p=0.006), *MYH4* (p=0.029)
38	17q21.2	-
39	18p11.23	-
40	19p13.2 (peak A) (n=7)	*OR7G1** (p=0.006), *MBD3L1* (p=0.035)
41	19p13.2 (peak B)	-
42	19q13.33	-
43	19q13.42 (n=4)	*NCR1* (p=0.047)
44	20q13.33	-
45	21q22.3	-
46	22q11.1	-

The number of genes found in each peak region is shown in parenthesis in the first column. Genes in regions whose expression were statistically correlated with the peak intensities are shown in the second column. The p-values shown in column 2 are

unadjusted. After Bonferroni correction, based on the number of genes tested within each region, the genes indicated with an asterisk remained statistically significant.

Supplementary Table 4. Amplification and deletion CNV peaks in TCGA and PHTS BC samples, showing peak regions and the genes which are contained within

Separate excel file.

Supplementary Table 5. Clinical characteristics of PHTS-related breast cancer series

Separate excel file.

Supplementary Table 6. CIBERSORT reveals significant differences in immune cell populations between PHTS-derived and TCGA BCs

Significantly increased cell population	*Increased group*	*P-value*
B cells naive	*PHTS*	*0.0003*
Dendritic cells resting	*TCGA*	*<0.0001*
Eosinophils	*TCGA*	*<0.0001*
Macrophages M0	*PHTS*	*<0.0001*
Macrophages M1	*TCGA*	*<0.0001*
Macrophages M2	*PHTS*	*<0.0001*
Mast cells	*TCGA*	*0.0004*
Mast cells resting	*PHTS*	*0.0014*
Monocytes	*PHTS*	*<0.0001*
Neutrophils	*TCGA*	*(0.373)*
NK cells activated	*PHTS*	*<0.0001*
T cells CD4 memory activated	*TCGA*	*<0.0001*
T cells CD8	*TCGA*	*0.0011*
T cells follicular helper	*TCGA*	*<0.0001*
T cells gamma delta	*TCGA*	*<0.0001*
T cells regulatory (Tregs)	*PHTS*	*<0.0001*

Comparison between PHTS-derived BCs and sporadic BCs from TCGA in the proportion of each cell population by t-test**.** For each cell population, the group (PHTS vs TCGA) which had increased proportion compared to the other group is indicated in column 2, along with the P-value in column 3.
