# Supplementary material for: Integrating somatic CNV and gene expression in breast cancers from women with PTEN hamartoma tumor syndrome
Source: NPJ Genom Med. 2023 Jul 5;8:14. doi: 10.1038/s41525-023-00361-0 (PMC10322985; doi:10.1038/s41525-023-00361-0)
Supplement: Supplementary file 4 — Reporting Summary [file 41525_2023_361_MOESM4_ESM.pdf]

nature portfolio

Corresponding author(s): Charis Eng, MD, PhD

Last updated by author(s): Apr 30, 2023

Reporting Summary

Nature Portfolio wishes to improve the reproducibility of the work that we publish. This form provides structure for consistency and transparency in reporting. For further information on Nature Portfolio policies, see our Editorial Policies and the Editorial Policy Checklist.

Statistics

For all statistical analyses, confirm that the following items are present in the figure legend, table legend, main text, or Methods section.

n/a	Confirmed	
☐	☒	The exact sample size (*n*) for each experimental group/condition, given as a discrete number and unit of measurement
☐	☒	A statement on whether measurements were taken from distinct samples or whether the same sample was measured repeatedly
☐	☒	The statistical test(s) used AND whether they are one- or two-sided<br>*Only common tests should be described solely by name; describe more complex techniques in the Methods section.*
☒	☐	A description of all covariates tested
☐	☒	A description of any assumptions or corrections, such as tests of normality and adjustment for multiple comparisons
☐	☒	A full description of the statistical parameters including central tendency (e.g. means) or other basic estimates (e.g. regression coefficient) AND variation (e.g. standard deviation) or associated estimates of uncertainty (e.g. confidence intervals)
☐	☒	For null hypothesis testing, the test statistic (e.g. *F*, *t*, *r*) with confidence intervals, effect sizes, degrees of freedom and *P* value noted<br>*Give P values as exact values whenever suitable.*
☒	☐	For Bayesian analysis, information on the choice of priors and Markov chain Monte Carlo settings
☒	☐	For hierarchical and complex designs, identification of the appropriate level for tests and full reporting of outcomes
☒	☐	Estimates of effect sizes (e.g. Cohen's *d*, Pearson's *r*), indicating how they were calculated

Our web collection on statistics for biologists contains articles on many of the points above.

Software and code

Policy information about availability of computer code

Data collection	No software was used to collect PHTS clinical data. TCGA data originated from the GDC data portal (open source).
Data analysis	FACETS (version 0.5.6), gistic2 (version 6.15.28), trimmomatic (version 0.39), BBmap (version 37.96), STAR (version 2.7.8), DESeq2 (version 1.34.0), pheatmap (version 1.0.12), EnhancedVolcano (version 1.12.0), R (version 4.1.2), GraphPad Prism (version 9.0), maftools (version 2.10.0).

For manuscripts utilizing custom algorithms or software that are central to the research but not yet described in published literature, software must be made available to editors and reviewers. We strongly encourage code deposition in a community repository (e.g. GitHub). See the Nature Portfolio guidelines for submitting code & software for further information.

Data

Policy information about availability of data

All manuscripts must include a data availability statement. This statement should provide the following information, where applicable:

- Accession codes, unique identifiers, or web links for publicly available datasets
- A description of any restrictions on data availability
- For clinical datasets or third party data, please ensure that the statement adheres to our policy

Due to IRB restrictions, the datasets used and/or analyzed during the current study are available from the corresponding author on reasonable request.

Research involving human participants, their data, or biological material

Policy information about studies with human participants or human data. See also policy information about sex, gender (identity/presentation), and sexual orientation and race, ethnicity and racism.

Reporting on sex and gender	Used the term sex (biological attribute) only. Sex was determined based on the medical record. Only female samples were used for this study. Thus, no sex-based analyses were performed.
Reporting on race, ethnicity, or other socially relevant groupings	No socially constructed/relevant data were included in the study.
Population characteristics	Population characteristics include: age of breast cancer diagnosis, breast cancer histologic subtype, breast cancer stage, tumor markers, CC score, genotype, intrinsic subtype (PAM50).
Recruitment	Females with diagnosis of PTEN hamartoma tumor syndrome were recruited under the IRB protocol 8458. No self-selection bias.
Ethics oversight	The Cleveland Clinic IRB approved the protocol.

Note that full information on the approval of the study protocol must also be provided in the manuscript.

Field-specific reporting

Please select the one below that is the best fit for your research. If you are not sure, read the appropriate sections before making your selection.

☒ Life sciences ☐ Behavioural & social sciences ☐ Ecological, evolutionary & environmental sciences

For a reference copy of the document with all sections, see nature.com/documents/nr-reporting-summary-flat.pdf

Life sciences study design

All studies must disclose on these points even when the disclosure is negative.

Sample size	We performed sample size calculations to determine the minimum number of cases we need to be powered to identify statistically significant genomic differences between the PHTS and TCGA sporadic BC groups. In order to detect characteristic differences at the variant level, we used the two proportions derived from the somatic PTEN mutation rate in the preliminary PHTS group with 29 samples (21.0%) and that of sporadic luminal subtypes in the literature (4.0%). We estimated that 30 samples from PHTS and 250 samples from TCGA should be sufficient to achieve a power of 81.0% with an alpha of 0.05 (two-sided) to detect a significant difference. RNA was extracted from samples with sufficient tissue materials.
Data exclusions	Exclusion criteria include: male samples, PTEN polymorphisms, breast caner cases whose tissue samples are not available
Replication	No other replication sample set for PHTS-derived BC is available because of the rarity of the condition (we work with the largest series of PHTS patients worldwide)
Randomization	Experimental group is made up of PHTS-derived breast cancer samples (with germline PTEN mutations). The control group consists of sporadic breast cancer samples (no germline mutations in breast cancer susceptibility genes).
Blinding	No blinding due to the nature of the study

Reporting for specific materials, systems and methods

We require information from authors about some types of materials, experimental systems and methods used in many studies. Here, indicate whether each material, system or method listed is relevant to your study. If you are not sure if a list item applies to your research, read the appropriate section before selecting a response.

Materials & experimental systems

n/a	Involved in the study
☒	☐ Antibodies
☒	☐ Eukaryotic cell lines
☒	☐ Palaeontology and archaeology
☒	☐ Animals and other organisms
☐	☒ Clinical data
☒	☐ Dual use research of concern
☒	☐ Plants

Methods

n/a	Involved in the study
☒	☐ ChIP-seq
☒	☐ Flow cytometry
☒	☐ MRI-based neuroimaging

Clinical data

Policy information about clinical studies

All manuscripts should comply with the ICMJE guidelines for publication of clinical research and a completed CONSORT checklist must be included with all submissions.

Clinical trial registration	This study is not a clinical trial
Study protocol	Study protocol is available under the Cleveland Clinic IRB protocol 8458.
Data collection	Approved by the Cleveland Clinic's Institutional Review Boards (IRB), written informed consents were obtained from all ndividuals enrolled under the study protocol. Among 6934 research participants prospectively accrued from September 1, 2005 to September 10, 2020, we identified 3066 female participants with a personal history of breast cancer (BC). Of these, 130 had germline PTEN variants. We then identified 44 women with appropriate consents for acquisition of biospecimens and whose tissues representing BC were available for sequencing.
Outcomes	There are statistically significant differences at the genomic level between the PHTS and TCGA sporadic breast cancer groups
